# Inhomogeneous Magnetic Anisotropy in an Fe_5–*x*_GeTe_2_ Nanoflake Observed by Imaging

**DOI:** 10.1021/acsnano.5c07836

**Published:** 2025-07-07

**Authors:** Massimo Ghidini, Vladimir Farenkov, Yang Li, Peter J. Newton, Raffaele Pellicelli, Samer Kurdi, Nadia A. Stelmashenko, Francesco Maccherozzi, Crispin H. W. Barnes, Andrew F. May, Manish Chhowalla, Sarnjeet S. Dhesi, Neil D. Mathur

**Affiliations:** † Department of Mathematics, Physics and Computer Science, 9370 University of Parma, 43124 Parma, Italy; ‡ 120796 Diamond Light Source, Chilton, Didcot, Oxfordshire OX11 0DE, U.K.; § Department of Materials Science, 2152 University of Cambridge, Cambridge CB3 0FS, U.K.; ∥ Cavendish Laboratory, University of Cambridge, Cambridge CB3 0US, U.K.; ⊥ Zernike Institute for Advanced Materials, University of Groningen, 9747 AG Groningen, The Netherlands; # Institute of Photonics and Quantum Sciences, SUPA, Heriot-Watt University, Edinburgh EH14 4AS, U.K.; ∇ Materials Science and Technology Division, 6146 Oak Ridge National Laboratory, Oak Ridge, Tennessee 37831, United States

**Keywords:** 2D materials, FGT, nanoflake, spintronics, imaging, PEEM, XMCD

## Abstract

Few-layer flakes of ferromagnetic Fe_5–*x*_GeTe_2_ with *x* = 0.3 (F5GT) possess a *c*-axis magnetocrystalline anisotropy that is large enough below ∼200 K to outcompete the easy-plane shape anisotropy, yielding distinctive magnetic microstructures with out-of-plane (OOP) magnetizations. Using photoemission electron microscopy (PEEM) with magnetic contrast from X-ray magnetic circular dichroism (XMCD) to study a thermally demagnetized h-BN-protected nanoflake of F5GT at 110 K, we observe a micron-scale coexistence between domains with OOP magnetizations (∼70% areal fraction) and hitherto unknown domains in which in-plane (IP) magnetization components dominate (∼30% areal fraction). The regions with dominant IP magnetization components do not correlate with small variations of flake thickness (6–10 nm) and instead arise from local changes of magnetocrystalline anisotropy due to a hitherto unidentified chemical inhomogeneity that we suggest to be a higher concentration of Fe vacancies. Our observation of micron-scale inhomogeneity would likely be missed if imaging a single flake orientation and should affect the viability and performance of van der Waals (vdW) spintronic devices with F5GT electrodes.

## Graphical abstract

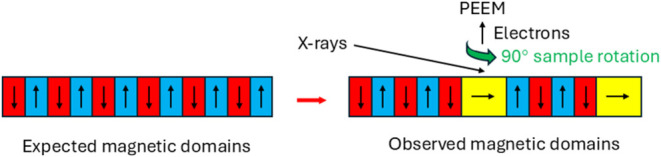

## Introduction

The recent discovery of long-range magnetic order in unit cell-thick layers of chromium germanium telluride (Cr_2_Ge_2_Te_6_)^1^ and chromium triiodide (CrI_3_)^2^ has both revitalized the field of two-dimensional (2D) magnetism^3^ and added 2D magnets such as these and Fe_5–*x*_GeTe_2_ ^4^ to the expanding library of 2D materials.^5^ By exploiting the exfoliation and integration techniques developed for nonmagnetic vdW materials,^6,7^ it has been possible to tune magnetic properties by varying flake thickness^5,8^ and to integrate magnetic flakes with other vdW materials for new functionality.^5,7^ For example, one may use vdW materials as room-temperature spintronic electrodes and perhaps exploit proximity effects,^9^ tune long-range magnetic order via layer number,^10^ stabilize Moiré magnetism in twisted-layer stacks,^11^ achieve homophase^12^ or heterophase^13^ multiferroicity, and tune the anomalous quantum Hall effect.^14^

Bulk Fe_5–*x*_GeTe_2_ is a metallic ferromagnet that adopts the rhombohedral structure of its *x* = 1 end member Fe_4_GeTe_2_, where *x* is the concentration of Fe vacancies.^15^ Fe_5–*x*_GeTe_2_ with *x* = 0.3 (F5GT) has a Curie temperature of 270 K < *T*_C_ < 310 K,^4^ and displays a complex magnetic behavior that is influenced by structural disorder and thermal history.^4,16–19^ For example, quenched crystals are metastable and display a first-order magnetostructural transition near 100 K.^4^ In F5GT flakes below ∼200 K, there is a *c*-axis perpendicular magnetic anisotropy (PMA).^4,16,17,20^ The PMA tends to outcompete thickness-dependent IP shape anisotropy to yield a rich variety of magnetic structures with OOP magnetizations, e.g., stripes that often adopt labyrinthine structures, fractal domains, and bubbles.^21–25^ In flakes and much thicker samples of F5GT, reports of IP magnetization either fail to consider the demagnetizing factor in *c*-axis magnetization measurements,^19^ or rely upon the IP application of 30 mT to overcome the OOP anisotropy field, albeit without annihilating the complex magnetic structures that result, e.g., merons.^24^

Here we present XMCD-PEEM images of an h-BN-protected, thermally demagnetized F5GT flake (*x* = 0.3) at 110 K, down to which we find no evidence for the magnetostructural transition that can arise near this temperature.^4^ Image contrast identifies the projection of the local magnetization on the grazing-incidence X-ray beam.^26^ For some micron-scale regions, 90° sample rotation about the *c*-axis has little effect on the image, evidencing domains whose magnetizations lie parallel/antiparallel to the *c*-axis due to the PMA.^4,17,20^ For other micron-scale regions, this 90° rotation substantially modifies the image, evidencing domains with a substantial uniaxial IP magnetization component. The distribution of the two types of region does not correlate with small variations in flake thickness (6–10 nm). Instead, we suggest that the substantial IP magnetization components arise in regions where the PMA is reduced by higher concentrations of Fe vacancies.^16,24^ More generally, one may anticipate that the PMA, magnetization and Curie temperature of F5GT will all be reduced when the concentration of Fe vacancies is increased, given that the *x* = 1 end member Fe_4_GeTe_2_ has IP anisotropy and a reduced Curie temperature.^15^ Similar changes are observed in the related but structurally different compound Fe_3–*x*_GeTe_2_.^16^

By performing the rotation study and finding that the OOP magnetic domains are accompanied by IP magnetic domains, we identify a hitherto unknown complexity in FGT on the micron scale. The presence of IP domains affects the performance of F5GT nanoflakes in spintronics, suggesting that imaging of the type we perform here^26,27^ represents an important form of hitherto overlooked characterization.

## Results

The F5GT nanoflake of interest is essentially triangular (dark-gray region, Figure 1a). The h-BN (dotted red line, Figure 1b) protects a region displaying steps (dotted black lines, Figure 1b) that separate terraces whose heights differ by 1–2 layers (white numbers in Figure 1b give both layer numbers and nm thicknesses). Four topographical XAS-PEEM images, obtained for a single sample orientation, were combined to show most of the h-BN-protected flake (bright regions in Figure 1c). Dark regions in Figure 1c either lie outside the flake or contain unprotected regions of flake that have oxidized/deteriorated.

**Figure 1:**
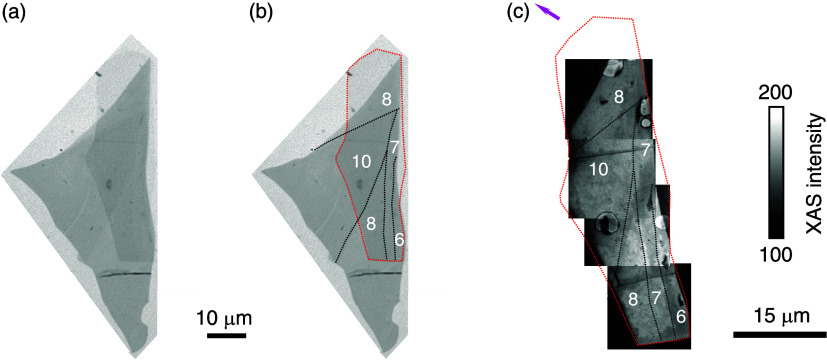
The h-BN-protected F5GT nanoflake. (a) Optical microscopy image of the triangular nanoflake (dark gray) and nearby background (light gray border). (b) The same image with overlays highlighting h-BN (dotted red outline) and terrace steps (black dotted lines). White numbers represent both layer number and flake thicknesses in nm. (c) Composite XAS-PEEM image showing most of the protected nanoflake, overlays copied from (b). Pink arrow shows IP projection of grazing-incidence X-ray beam.

Figure 2a,b shows composite XAS-PEEM images for our two orthogonal sample orientations (Figure 2b represents a repeat of Figure 1c). The two composite XAS-PEEM images are nominally equivalent to each other, but in practice they differ slightly due to distortions arising from the PEEM lenses. As a consequence of these distortions, there is some mismatch between the colored outlines that we have introduced in Figure 2a, 2b to identify the regions on which we will zoom in Figures 3–6. Figure 2c,d shows magnetic domains (red and blue) in the two corresponding composite XMCD-PEEM images, which taken together represent the hitherto unknown magnetic inhomogeneity of interest even without zooming-in. The top and bottom regions of the two XMCD-PEEM images show that sample rotation produces little change, implying that the local magnetization lies OOP due to strong PMA, as expected.^4,16,20^ However, the central regions of the two XMCD-PEEM images show that sample rotation produces a change of contrast, implying that an IP component of magnetization dominates locally.

**Figure 2:**
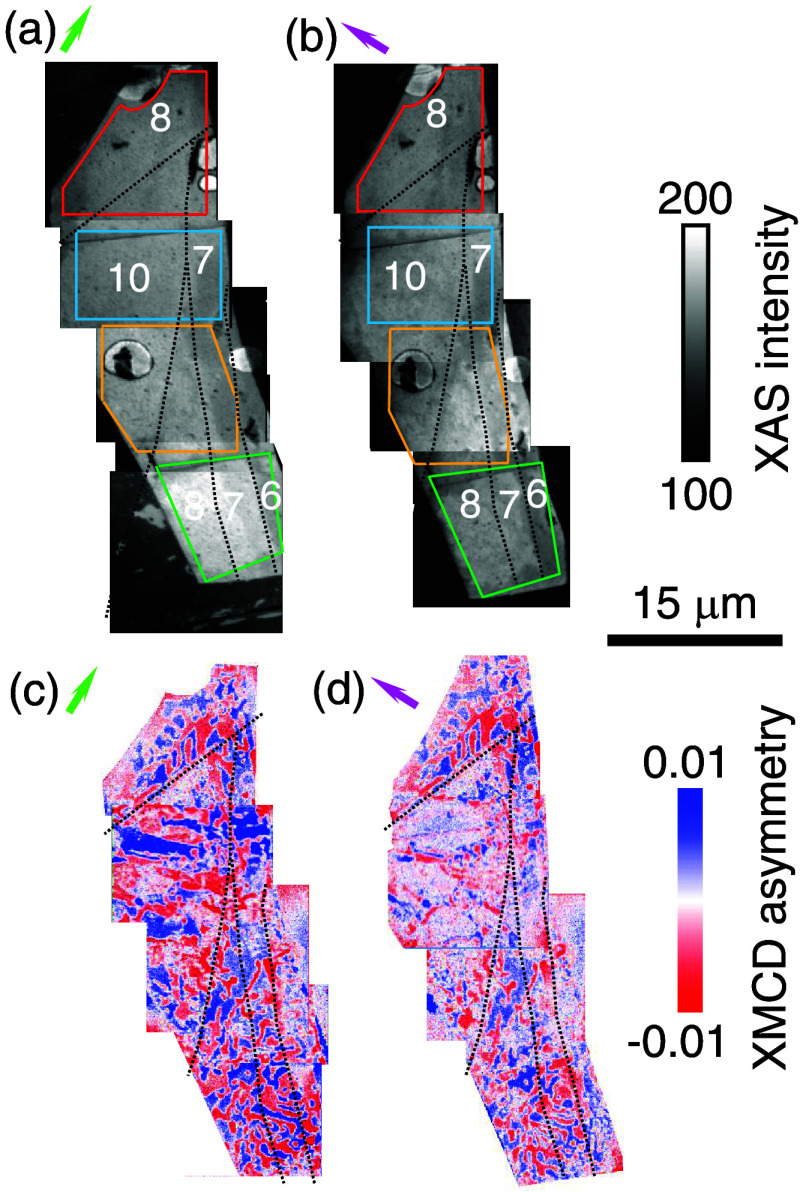
Composite images of the h-BN-protected F5GT nanoflake. Each panel shows four overlapping 15 μm × 15 μm images obtained by (a, b) XAS-PEEM and (c, d) XMCD-PEEM. The IP projection of the grazing-incidence X-ray beam is identified via the green and magenta arrows. Panel (b) repeats the data shown in Figure 1c. All panels repeat the terrace steps (black) shown in Figure 1b, c. Colored outlines in (a, b) approximately identify the XMCD-PEEM images in panels (a, b) of Figure 3–6

**Figure 3:**
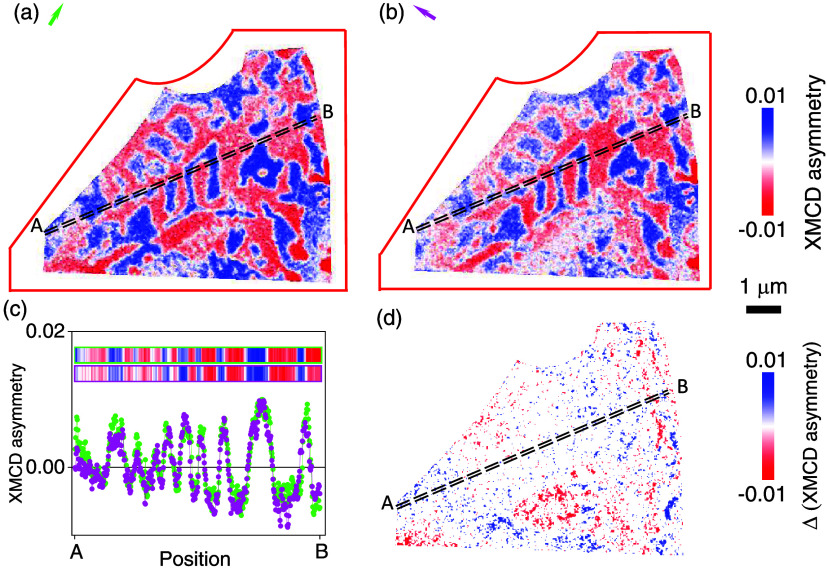
Details of magnetic structure for the red-outlined region in Figure 2a, b. (a, b) Zooms of the XMCD-PEEM images in Figure 2(c, d) for approximately this region, with color outlines expanded for clarity. (c) XMCD asymmetry along A–B transect between dashed black lines in (a), shown as vertically stretched pixels in green outline and also as green data points; and XMCD asymmetry along the A–B transect between dashed black lines in (b), shown as vertically stretched pixels in magenta outline and also as magenta data points. (d) Difference image (a, b) with values below |0.004| set to zero.

The regions with dominant IP components of magnetization do not correlate with the observed thickness variations, which are too small to substantially modify the demagnetizing factor (Figure 2b). Instead, we may infer that shape anisotropy dominates a PMA that is locally reduced by higher concentrations of Fe vacancies.^15,16^ These regions with dominant IP magnetization components occupy roughly 30% of the magnetically imaged flake (dissimilar regions in Figure 2c,d).

We will now consider the XMCD-PEEM data in more detail by focusing in Figures 3–6 on the regions in Figure 2a,b that are roughly outlined red, blue, yellow and green. XMCD-PEEM data for the red-outlined region show little change following the 90° rotation (Figure 3): the images showing magnetic domains in Figure 3a,b are similar to each other, as shown explicitly by comparing A–B transects (Figure 3c), and by presenting a difference image in which many regions are equivalent (white in Figure 3d). Thus, the red-outlined region primarily comprises domains whose magnetizations lie along the *c*-axis, but small, nonzero regions in the difference image evidence small, disconnected regions with a dominant IP magnetization component, implying local reduction of PMA.

XMCD-PEEM data for the blue-outlined region show changes in some regions but not others following the 90° rotation (Figure 4). The C–D transects through Figure 4a,b are similar nearer C and different nearer D (Figure 4c), while the E–F transects are similar all along (Figure 4d). Overall, the difference image (Figure 4e) contains large nonzero regions, e.g., around the C–D transect nearer D, where a predominance of head-to-head IP magnetization components lie parallel and antiparallel to the green arrow in Figure 4a, thus identifying an IP easy axis. The magnetostatic energy cost of the head-to-head configuration implies metastability, perhaps consistent with the metastability identified in quenched crystals,^17^ where uniaxial IP anisotropy could be associated with either higher Fe vacancy concentrations that suppress PMA, or strain from exfoliation, transfer, or interaction with the substrate.^30^

**Figure 4:**
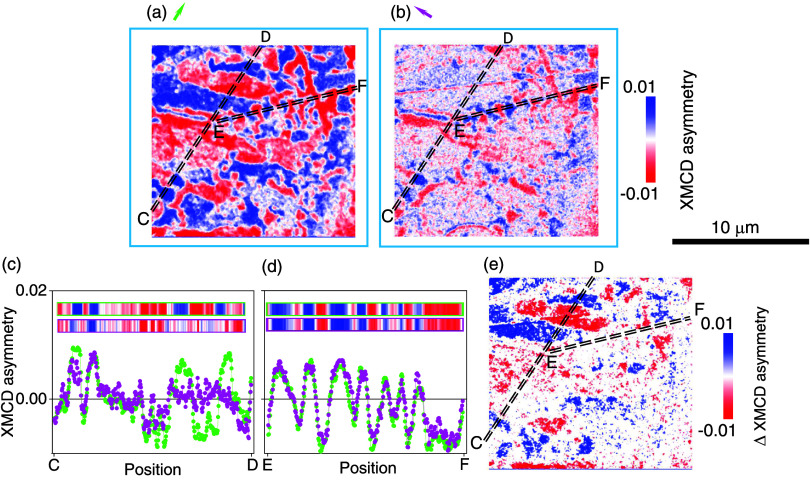
Details of magnetic structure for the blue-outlined region in Figure 2a,b. Caption as for Figure 3 except second transect yields extra panel.

XMCD-PEEM data for the orange-outlined region primarily show changes top-left following the 90° rotation (Figure 5). These changes are explicitly shown via the G–H transect (Figure 5c,e), as before arising due to large head-to-head IP magnetization components (top-left in Figure 5a,b). Other regions show little change, e.g., along the I–J transect (Figure 5d,e), evidencing the expected OOP local magnetization.^4,16,20^

**Figure 5:**
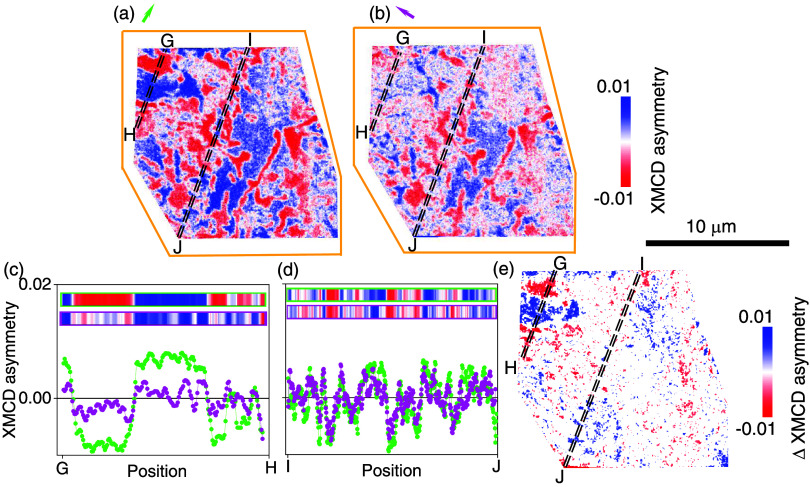
Details of magnetic structure for the yellow-outlined region in Figure 2a,b. Caption as for Figure 3 except second transect yields extra panel.

XMCD-PEEM data for the green-outlined region show that the 90° rotation produces relatively small changes of intensity, such that the same pattern of domains is readily apparent for each orientation in the images (Figure 6a,b) and their transects (Figure 6c,d). The similarity of the difference image (Figure 6e) with respect to both parent images (Figure 6a,b) suggests a homogeneous canting of magnetization due to a homogeneous reduction of PMA. Much of the green-outlined region is therefore somewhat anomalous with respect to the rest of the examined flake.

**Figure 6:**
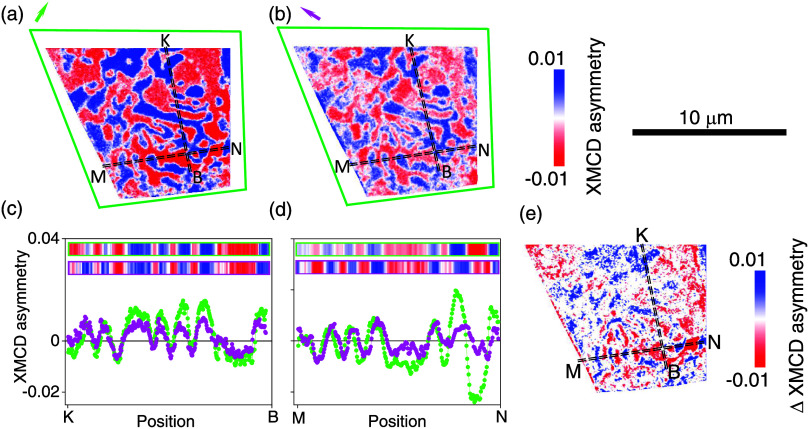
Details of magnetic structure for the green-outlined region in Figure 2a,b. Caption as for Figure 3 except second transect yields extra panel.

## Conclusions

We have shown that XMCD-PEEM images for micron-scale regions in our F5GT nanoflake at 110 K are affected differently by 90° sample rotation. Regions that show little change contain the expected domains possessing OOP magnetizations^4,16,20^ (∼70% areal fraction). Other regions that show large changes contain magnetic domains whose magnetizations lie wholly or partially IP (∼30% areal fraction). In many of the latter regions, the fortuitous alignment of the IP magnetization component with the IP projection of the X-ray beam prior to 90° rotation, and the reduction of the XMCD asymmetry to near zero after 90° rotation, suggest the local magnetization may lie almost wholly in-plane.

We suggest that the regions with strong IP components of magnetization possess higher concentrations of Fe vacancies, as a higher Fe vacancy concentration in Fe_5–*x*_GeTe_2_ is likely to reduce the PMA (cf. Fe_4_GeTe_2_ ^15^) and perhaps introduce an IP anisotropy^15,16^ (that could also arise from strain). The likely concomitant reduction of magnetization^15,16^ is recognized in the micromagnetic simulation that we present below, where plausible materials parameters permit the experimentally observed richness to be recreated.

We model a region of the F5GT nanoflake (Figure 7a) by assuming PMA around a micron-sized inclusion with uniaxial IP anisotropy instead of PMA, and reduced magnetization (the IP easy axis lies at 63° to our *x*-axis in order to mimic our experimental setup). The model yields a three-dimensional (3D) vector map (Figure 7b) in which perpendicular stripe domains populate the region with PMA, while two IP domains populate the inclusion. Projections of the magnetization along suitably chosen orthogonal IP directions yield maps (Figure 7c,d) that represent well some of our XMCD-PEEM images (Figures 4a,b and 5a,b), where sizable regions containing domains with strong IP components of magnetization coexist with sizable regions containing domains with OOP magnetizations. Note that both the OOP and IP magnetizations we simulated have a finite projection on the X-ray beam direction, except for the IP magnetizations that lie perpendicular to the beam direction (white in Figure 7d).

**Figure 7:**
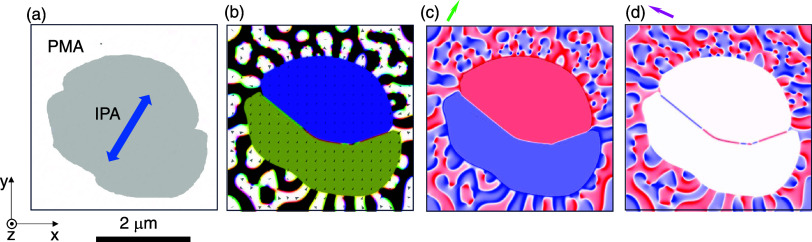
(a) Micromagnetic simulation of part of the F5GT nanoflake. (a) Material with strong PMA surrounds a micron-sized inclusion with reduced magnetization and uniaxial IP anisotropy (IPA, blue arrow identifies easy axis). (b) 3D vector map of magnetization. Black and white represent up and down OOP domains; blue and gold represent head-to-head domains with IP magnetizations along the IPA easy axis. (c, d) Simulated XMCD-PEEM images of the type we obtained (c) before and (d) after 90° sample rotation about the OOP *c*-axis. Data (*M*_projection_/*M*_s_) represent projection of normalized magnetization along the direction corresponding to the grazing-incident beam, whose IP projection is arrowed. Color scale ranges from red (*M*_projection_/*M*_s_ = −1) to white (*M*_projection_/*M*_s_ = 0) to blue (*M*_projection_/*M*_s_ = +1).

The experimentally observed head-to-head IP domains could not be reproduced with standard minimization procedures, as expected given their metastability. However, we show (Figure 7b–d) that such a configuration remains stable if initially set head-to-head with a magnetization of 200 kA m^–1^ (40% that of the OOP domains) and a uniaxial IP anisotropy *K*_IP_ ≥ 100 kJ m^–3^. An IP anisotropy of this magnitude could be either created by the chemical disorder we suggest, or by a small local strain of ε ∼ 0.5% given the large spontaneous magnetostriction λ of F5GT. For this strain calculation, $K_{\mathrm{IP}}=\frac{3}{2}E\epsilon \lambda$ ,^25^ we assume the Fe_3_Ge Young’s modulus of *E* = 170 GPa,^31^ and we assume a value of λ = 100 ppm that is half the measured magnetostriction for bulk Fe_3_GeTe_2_ at *T* = 100 K.^32^

It is hard to see how the proposed variations of Fe vacancy concentration could be resolved either locally or globally. However, it would be interesting to investigate how the observed magnetic structures evolve with temperature and thermal cycling in F5GT nanoflakes of different thickness. More generally, the possibility of magnetic inhomogeneity in F5GT or similar such materials should be considered when fabricating and analyzing spintronic devices, preferably by magnetically imaging magnetic electrodes, or at least the very material that is used to fabricate magnetic electrodes. For example, F5GT electrodes were recently inferred to possess a spin polarization with both IP and OOP components that affect spin injection.^33^ More generally, direct evidence from imaging, of the type we provide here, should be exploited as a useful diagnostic for investigating and improving the performance of spintronic devices.

## Methods

### Sample Fabrication

Variable-thickness F5GT flakes were prepared by mechanical cleavage of a single crystal that was grown by chemical vapor transport and subsequently quenched. The vendor (HQ graphene) used energy dispersive X-ray analysis (EDX) to identify a composition of Fe_4.7_GeTe_2_, i.e., F5GT with *x* = 0.3. The vendor also used SQUID magnetometry to identify a Curie temperature of ∼300 K. Protective h-BN flakes (∼5 nm thick) were also prepared by mechanical cleavage from a bulk single crystal (HQ graphene). F5GT flakes, and an h-BN flake over the single F5GT flake of interest, were transferred onto a metallized substrate with alignment markers within a glovebox (argon atmosphere, O_2_ and H_2_O content <0.1 ppm) using a dry-transfer system that included a micromanipulator, a hot plate and an optical microscope. The resulting sample was stored in the glovebox, transported to Diamond Light Source for XMCD-PEEM under vacuum, and exposed briefly to atmosphere during to transfer to the PEEM chamber.

The substrate, whose composition is not important here, was a single crystal of ferroelectric 0.68Pb­(Mg_1/3_Nb_2/3_)­O_3_-0.32PbTiO_3_ (011) (PMN–PT) from TRS, polished at CrysTec in order to reduce the peak-to-peak roughness from 300 nm to <1 nm, thus reducing the thickness from 0.8 to 0.35 mm. Metallization of the polished surface (to avoid charging in PEEM) was achieved by sputter depositing Ti­(1.5 nm) and then Pt­(25 nm). In order to find the F5GT flake in the PEEM chamber, 1 μm × 1 μm alignment markers of Ti­(6 nm)/Au­(20 nm) were thermally evaporated onto the top electrode via a lift-off mask prepared by e-beam lithography.

### XMCD-PEEM

All XMCD-PEEM data were obtained on I06 at Diamond Light Source, in zero applied magnetic field, after cooling from room temperature to 110 K. We used PEEM to map secondary-electron emission arising from circularly polarized X-rays that were incident on the sample surface at a grazing angle of 16°. The probe depth was ∼7 nm. Raw images were acquired during 1 s exposure times with right (R) and left (L) circularly polarized light that was tuned to the Fe *L*_3_ resonance at 708.3 eV. The pixels in a raw XMCD-PEEM image represent the projection of the local magnetization on the incident-beam direction: pixel intensity is given by the XMCD asymmetry (*I* ^R^ – *I* ^L^)/(*I* ^R^ + *I* ^L^), and *I* ^R/L^ denotes the intensity for secondary-electron emission due to X-ray absorption at the Fe *L*_3_ resonance. We averaged 200 raw XMCD-PEEM images to obtain one resultant XMCD-PEEM image for each of two orthogonal sample orientations. Difference images were then obtained via pixel-by-pixel subtraction. For the XMCD-PEEM images in Figures 3–6, and not the XMCD-PEEM images in Figure 2, this subtraction was performed after correcting for drift and distortion by using an affine transformation that was based on topographical XAS-PEEM images for each sample orientation (XAS is X-ray absorption). Each such XAS-PEEM image was obtained by averaging all of the raw images that had been obtained on resonance with left and right-polarized light.

### Micromagnetic Simulations

We used MuMax3.^28^ An F5GT volume of 4 μm × 4 μm × 8 nm was discretized into 2 nm × 2 nm × 8 nm cells. We assumed an exchange stiffness of 10^–11^ J m^–1^,^24^ a saturation magnetization of *M*_s_ = 500 kA m^–1^ and perpendicular anisotropy constant *K*_u_ = 160 kJ m^–3^ that reproduced the observed micron-sized OOP domains. An inclusion with *K*_u_ = 0, an IP anisotropy of *K*_IP_ = 100 kJ m^–3^, and a reduced magnetization of 200 kA m^–1^ yielded IP domains surrounded by OOP domains. Stable equilibrium configurations were identified via energy minimizations that exploited the conjugate gradient method, and these configurations were confirmed by dynamically relaxing the magnetization. From the resulting 3D maps of the magnetization, maps of the projection of the magnetization along the X-ray beam at an angle of 16° were constructed for direct comparison with the experimental XMCD-PEEM images. Note that our value of *K*_u_ = 160 kJ m^–3^ is similar to the value of *K*_u_ = 120 kJ m^–3^ that one obtains from a magnetic anisotropy energy of 0.021 meV/Fe,^29^ and that our exchange stiffness of 10^–11^ J m^–1^ is similar to first-principle values of 9.6 × 10^–12^ J m^–1^ and 24 × 10^–12^ J m^–1^.^20,29^
